# Percutaneous Thoracic Spinal Cord Stimulator Placement

**DOI:** 10.7759/cureus.13916

**Published:** 2021-03-16

**Authors:** Alison M Westrup, Andrew K Conner

**Affiliations:** 1 Department of Neurosurgery, University of Oklahoma Health Sciences Center, Oklahoma City, USA

**Keywords:** adult, operative report, pain management, percutaneous, prone position, spinal cord stimulation, technical report

## Abstract

Spinal cord stimulation is a safe, effective, and reversible method for the management of chronic neuropathic pain. Spinal cord stimulation was found to be superior to traditional conservative management in recent clinical trials. The superiority of this therapeutic strategy is in part due to the many benefits, such as decreased use of prescription pain medications, cost-effectiveness, and improvement in patient quality of life. With appropriate patient consent for photography during the operation per hospital policy, the technical description for percutaneous placement of a spinal cord stimulator was documented at the authors home institution. The percutaneous technique allows for decreased operative times and thus reduced anesthesia, as well as decreased post-operative pain due to less tissue and muscle dissection. Additionally, the percutaneous leads have a smaller footprint in the epidural space, allowing more patients with mild spinal canal stenosis to receive this therapeutic device, which generally precludes paddle placement. These features make the percutaneous method an appealing alternative to the traditional laminotomy technique. The traditional laminotomy approach for paddle lead placement has been well described in the literature. However, detailed and indexed techniques of the percutaneous alternative are lacking. This technical description provides the first, easily accessible technical guide for the percutaneous placement of thoracic spinal cord stimulators. The operative technique was documented with images and detailed descriptions at the authors home institution.

## Introduction

Spinal cord stimulation, a form of neuromodulation, has become an effective modality for treating chronic pain conditions since the 1960s [1,2]. A few of the many benefits of spinal cord stimulation are cost-effectiveness, improvement in quality of life, and decreased amounts of pain medications prescribed for patients [1,3]. The superiority of spinal cord stimulation compared with conservative treatment (particularly for failed back surgery syndrome) has been evidenced by two randomized controlled trials, detailing the effectiveness in pain improvement and functional outcomes [4,5].

Implantation techniques differ and depend largely on surgeon preference as well as institutional protocol [6]. This has historically been achieved by using a flat, two-dimensional insulated electrode, or paddle lead (lamitrode) placed via laminotomy. However, due to the invasiveness of the aforementioned technique, the percutaneous (octrode) or linear arrangement of conductors and insulators by a midline anchoring placement has become more popular [2,6]. Furthermore, the less invasive, percutaneous lead placement can typically be done in an outpatient setting, which reduces inherent risks associated with an extensive surgery (infection, duration of anesthesia, post-operative pain, etc.) [2]. In addition, the percutaneous leads have demonstrated improved durability or reduced risk of breakage compared to the alternative, laminotomy technique [2]. Historically, the laminotomy approach provided lower rates of lead migration; however, with the development of fascial anchors, the two techniques have comparable rates of migration [7].

This technique has been described briefly in textbooks or various novel approaches (i.e., patient in sitting position) [1], but it is not readily and rapidly accessible in peer-reviewed literature. Given that the literature is currently lacking a standard description of the operative technique for percutaneous thoracic spinal cord stimulation implantation, here we illustrate our institutional technique of percutaneous spinal cord stimulator placement.

## Technical report

**Figure 1:**
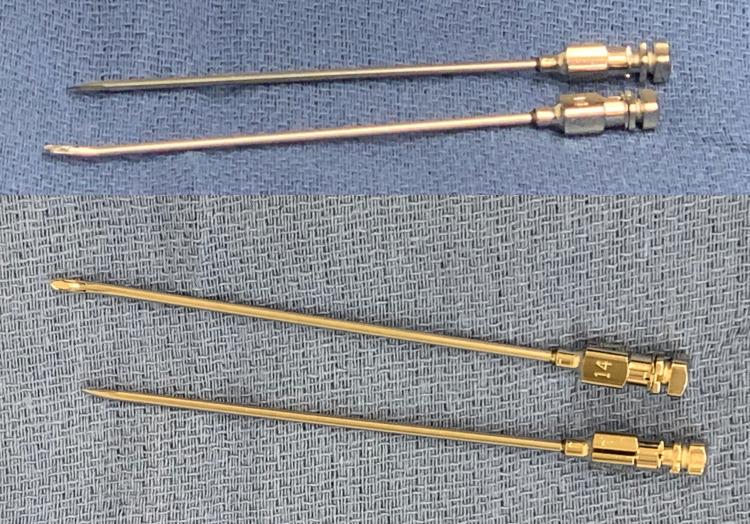
Tuohy needles. One needle has a straight tip and the other has a curved tip, best demonstrated by the top image.

**Figure 2:**
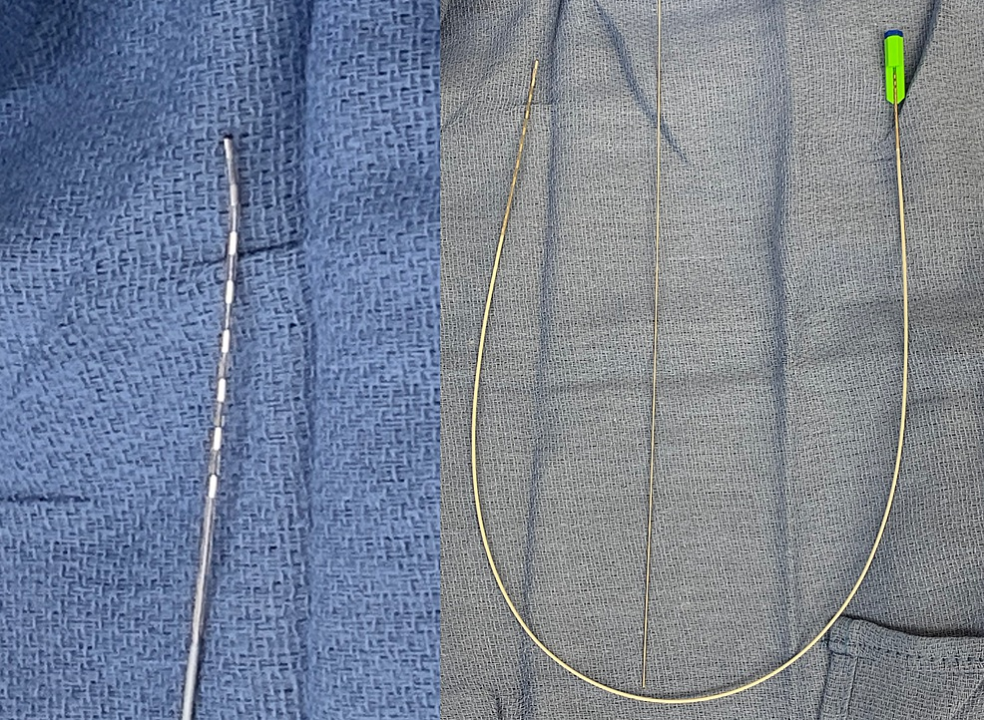
Electrodes. Left part of the image demonstrates the curved implantable electrode. The curve facilitates steering of the electrode in the epidural space. The kit also contains a straight electrode that is not pictured. Right part of the image shows the entire electrode wire with the capped tip, placed in the center is the guidewire.

The standard kit includes Tuohy needles (Figure 1), flexible guidewire, and percutaneous electrodes (Figure 2). Patient consent for photography during the operation was obtained with the appropriate documentation, per hospital policy.

The patient is placed under general anesthesia and neuromonitoring is utilized for the duration of the procedure. Somatosensory evoked potentials and free running electromyography are commonly utilized. The surgeon may choose to perform implantation under monitored anesthetic care with adjuvant local anesthesia to evaluate neurological status and pain region coverage during the procedure, or instead utilize information gathered from neuromonitoring with test stimulation, or even simply attempt to faithfully copy the patient’s successful spinal cord stimulator trial imaging without the use of other adjuncts. Pre-operative surgical prophylactic antibiotics are administered prior to the skin incision.

**Figure 3:**
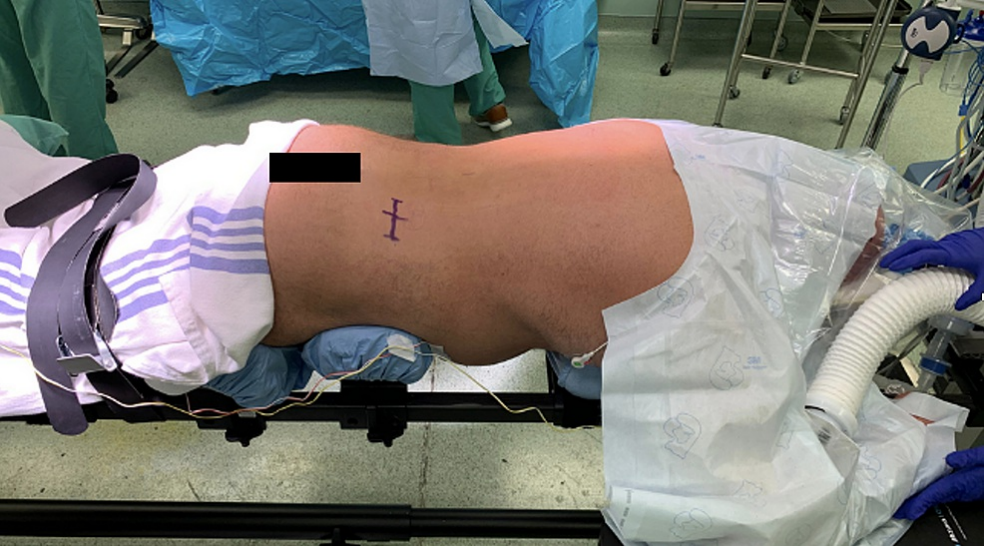
Patient positioning. The patient is positioned prone onto a Jackson table with hip and thigh pads. The arms are placed superiorly. The planned IPG incision is drawn two fingerbreadths below the posterior superior iliac spine. A Wilson or “Bow Frame” can also be used. IPG, implantable pulse generator

The patient is positioned prone onto a Jackson table with hip and thigh padding. A Wilson or “Bow-Frame” can also be utilized; however, one must be diligent regarding imaging alignment for final placement as this frame can cause imaging obtained to be misleading due to mechanically induced kyphosis. We have also found that these types of frames often have radio-opaque features that limit visualization. The patient’s arms are padded and placed flex to the side of the body (Figure 3).

The implantable pulse generator (IPG) incision site is agreed upon (laterality and location) in the pre-operative area. The incision is drawn at the desired location. Typical IPG locations include one to two fingerbreadths below the posterior superior iliac spine, significantly lateral from the midline to facilitate patient access to the device versus placement at the flank just below the last palpable rib, as well as significantly lateral from the midline as previously described.

**Figure 4:**
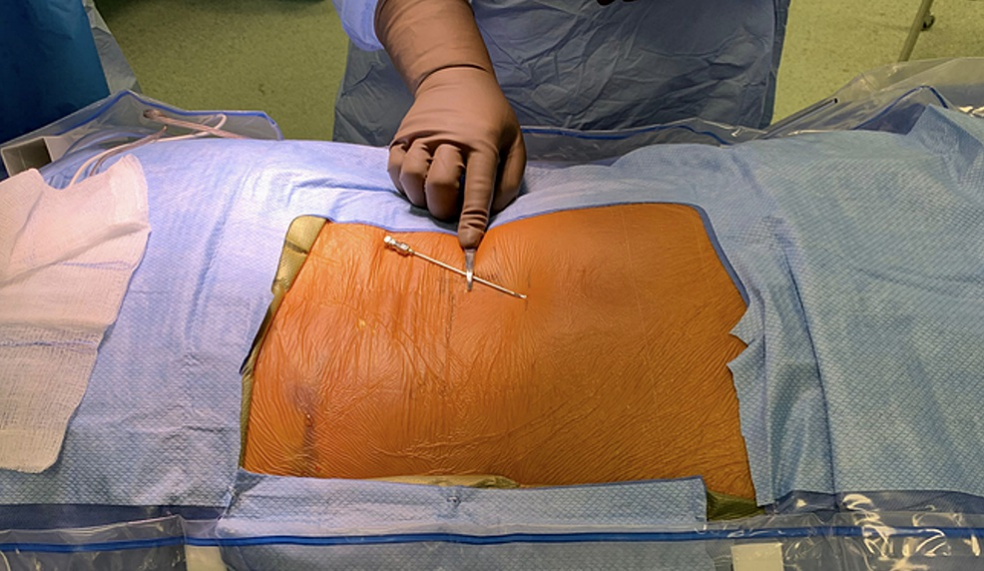
Incision planning. The provided 14-gauge curved Tuohy needle is utilized to plan the skin incision. The tip of the needle is moved to the proposed site of insertion, while the posterior end of the needle is place directly over the second pedicle below the insertion site and marked with another metallic instrument to designate the planned entry incision.

**Figure 5:**
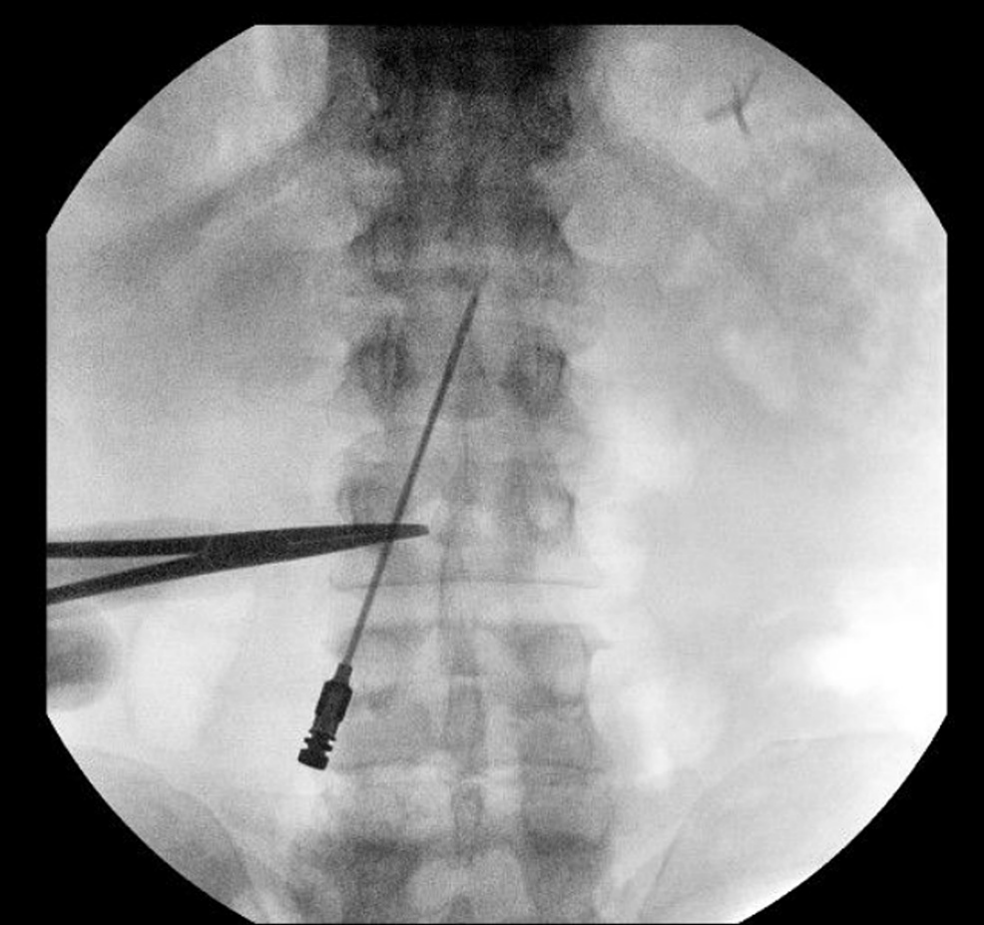
Incision planning confirmation. Fluoroscopy demonstrating the needle tip at L1-2 interspace, and tip of the metallic instrument over the second pedicle below insertion site to designate the planned entry incision.

The incision for the lead insertion site is determined by imaging. The typical entry site for percutaneous electrodes is the L1-2 interspace. Although other suitable locations, such as lower or higher interspaces, can be utilized, it is difficult due to the inherent anatomy of the thoracic spine to easily cannulate the epidural space above T12. A 14-gauge Tuohy needle is imaged with the tip overlying the point of intended epidural access (L1-2), and the distal end of the needle is placed approximately two pedicles below the entrance site (paramedian to either side) as this facilitates adequate needle trajectory into the epidural space (Figures 4, 5).

Some surgeons obtain epidural access with the Tuohy needle prior to skin incision; however, our practice is to create a skin incision prior to needle placement as this allows easier needle maneuverability, visualization of fascia, and in some patients facilitates identification of landmarks, such as the spinous process, via palpation through the small approximately 4 cm incision.

**Figure 6:**
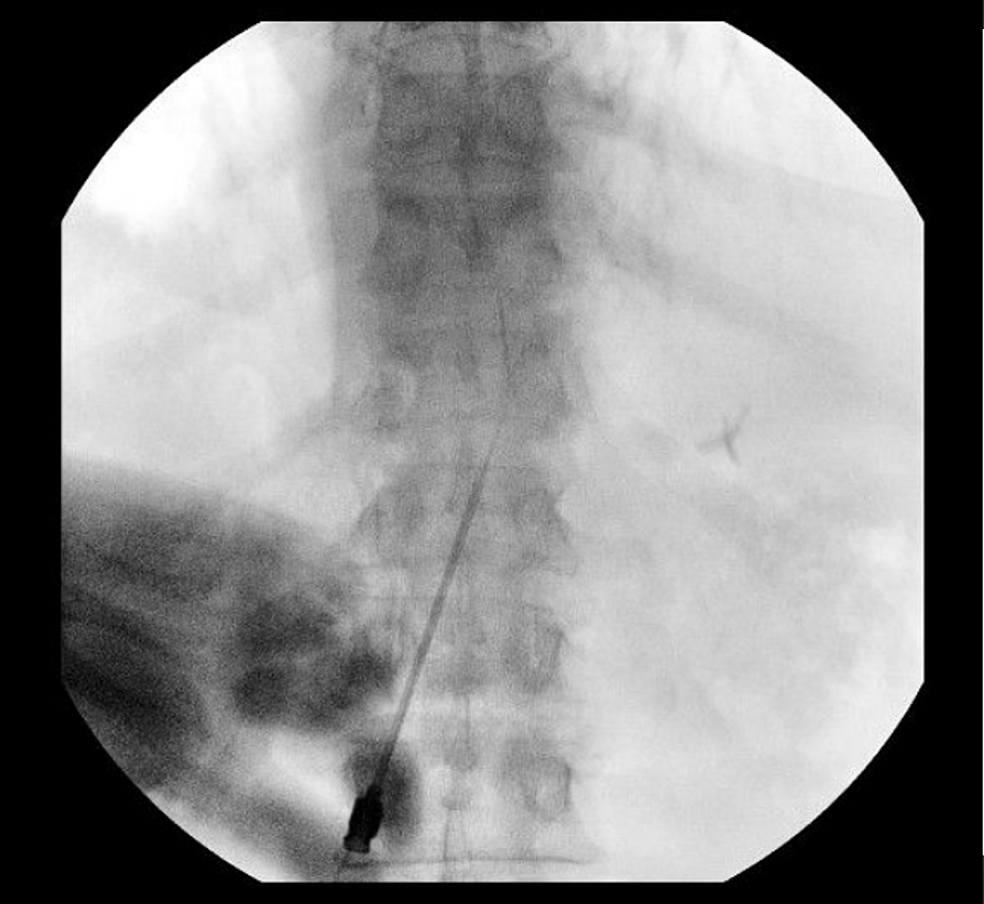
Epidural positioning. A soft guidewire is passed into the epidural space under fluoroscopy. If no resistance is met, this confirms appropriate epidural space positioning.

Once the incision is opened, the subcutaneous tissue is retracted to visualize the lumbodorsal fascia. The 14-gauge smooth curved Tuohy needle is then deployed towards the L1 spinous process until bony resistance is met. Intraoperative fluoroscopy is then utilized to confirm the level, and the needle is gently and slowly “walked down” the spinous process until the underside of the lamina is identified through tactile feedback. At this point, the inner stylet is removed. If cerebrospinal fluid is encountered, the needle should be withdrawn and re-deployed at the level above. The soft guidewire is passed through the Tuohy needle and should meet no resistance if the needle has accessed the epidural space (Figure 6).

The alternative method for confirming epidural placement is utilizing the “loss of resistance” technique, described as the passage of the epidural needle though the ligamentum flavum and feeling the decrease in resistance as the contents of a syringe can enter the epidural space easily [8]. Some practitioners utilize iodinated contrast to confirm epidural positioning. The lead is then driven into position. It is steerable and can be aided by the initial Tuohy needle bevel placement.

**Figure 7:**
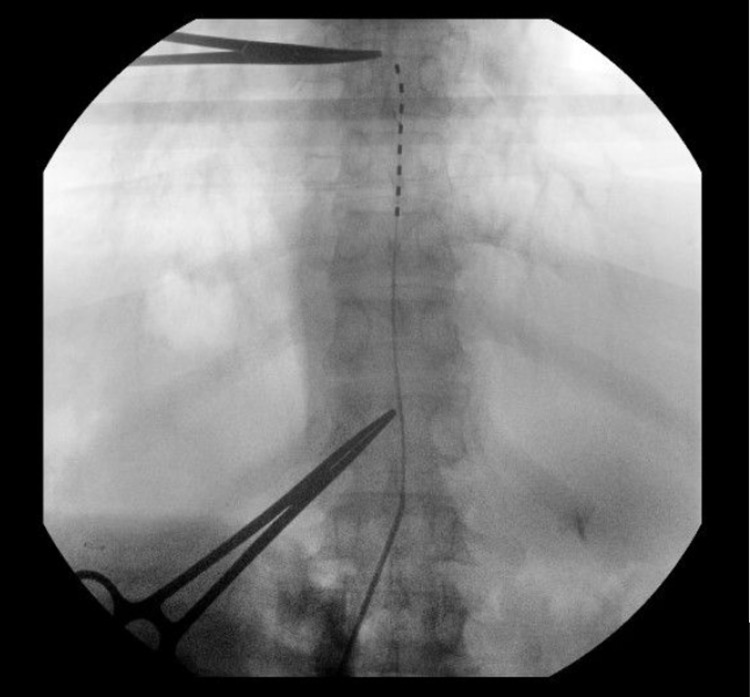
Electrode positioning. The percutaneous electrode is driven just past the intended target under live fluoroscopy. Here, the T12 pedicle and T7-8 disk space are marked with metallic instruments to simplify counting levels.

Typically, the first electrode is placed on the contralateral paramedian area of the dorsal spinal canal, hugging the spinous processes. Thus, the bevel of the needle can be turned to face away from the midline to help facilitate prior to deploying the electrode. It is not advisable to rotate the Tuohy needle in any way once the electrode has passed the tip as this can cause shearing of the wire. Once the lead is at the specified target, most commonly between T8-10, our practice is to overshoot the target by around half the distance of one vertebral body temporarily prior to final anchoring. (Figure 7).

**Figure 8:**
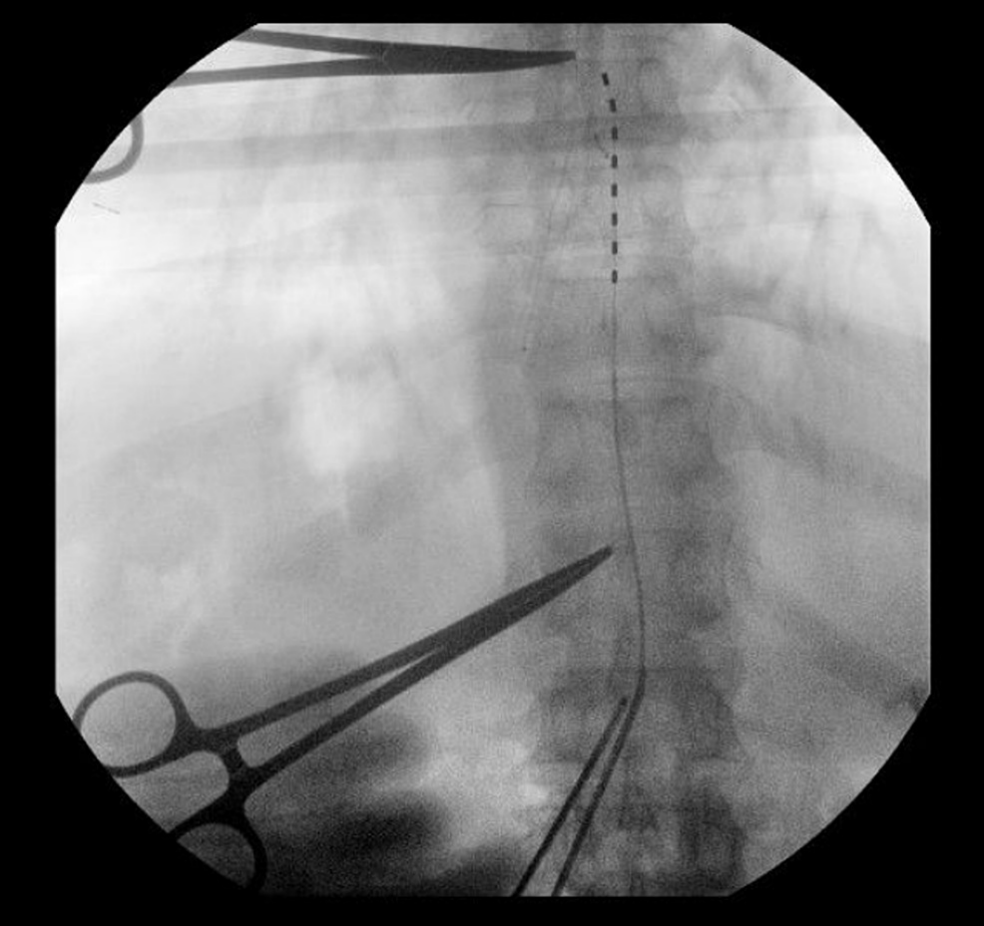
Electrode protection. A second 14-gauge Tuohy needle is deployed into the epidural space alongside the prior Tuohy needle and in the same fashion as the initial needle placement. The previous Tuohy needle is left deployed into the epidural space to “protect” the electrode wire from shearing that could potentially occur as a result of the second needle deployment.

The second Tuohy needle is then deployed lateral to the first in the same fashion (Figure 8).

**Figure 9:**
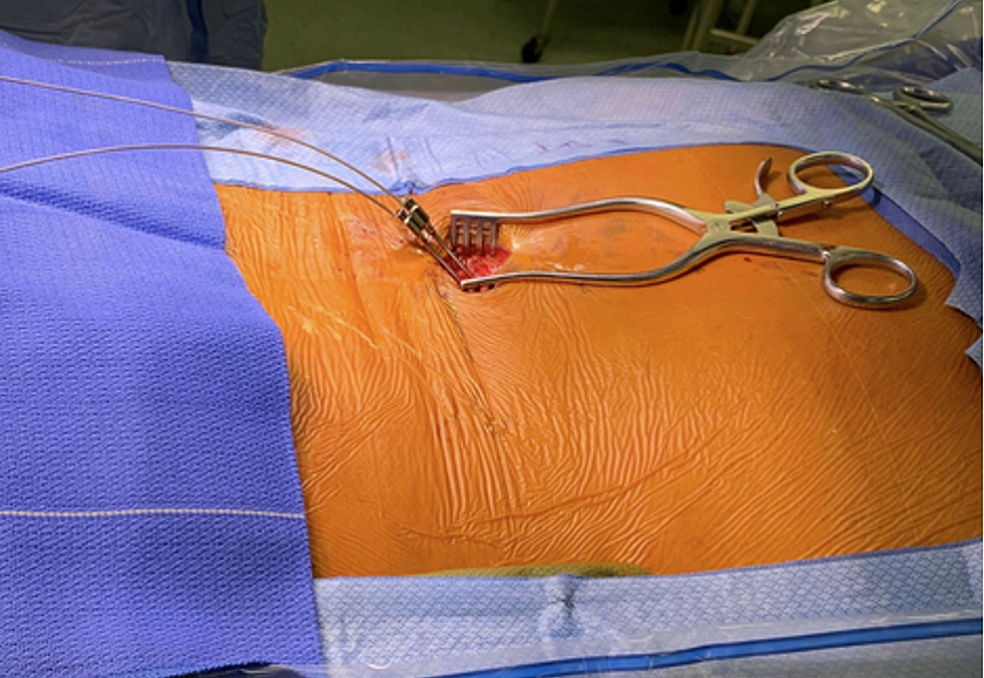
Electrode placement. The skin incision is made and each Tuohy needle is deployed into the epidural space under fluoroscopy. The percutaneous electrodes are then carefully driven into the intended position. A self-retaining retractor is seen here; however, the actual placement of the Tuohy needles and placement of the percutaneous electrodes is done without the retractor in place to facilitate imaging.

**Figure 10:**
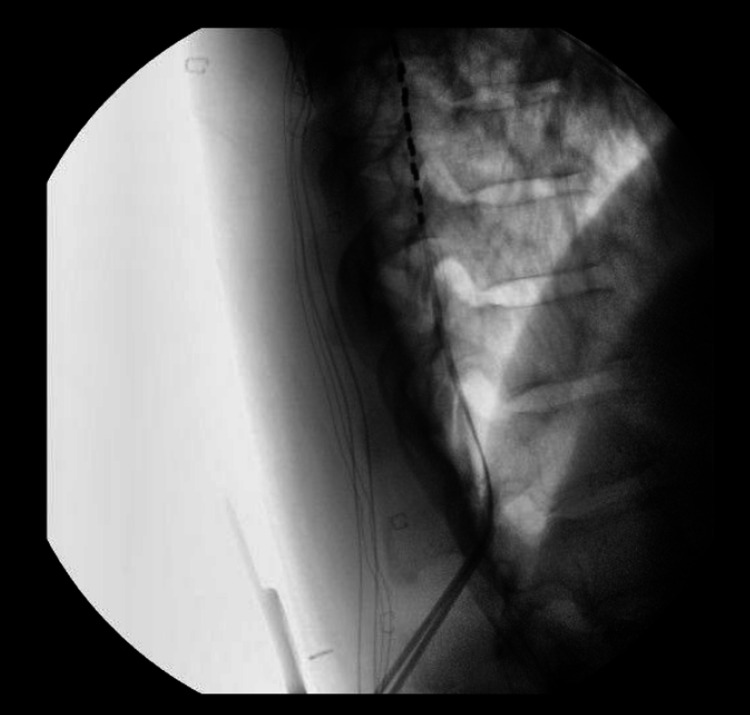
Epidural placement confirmation. Lateral fluoroscopy is obtained to ensure dorsal epidural placement prior to anchoring.

The prior needle is left “docked” to protect the other electrode from damage that could be caused by the second needle (Figures 9, 10).

**Figure 11:**
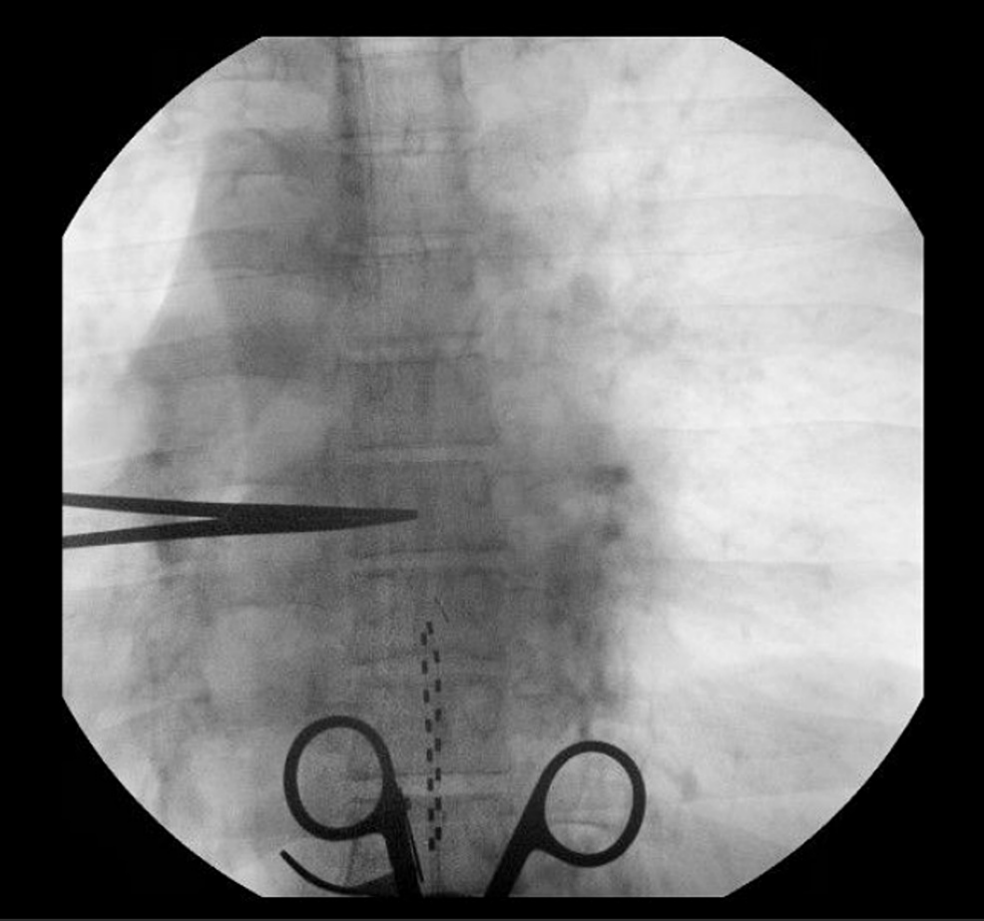
Final placement. Final lead placement is obtained by gently retracting the individual electrodes to the desired level. This tends to occur during anchoring naturally, hence, it is advisable to overshoot the electrode position initially prior to final positioning. It is also crucial to note that the end plates of the vertebral bodies are well aligned demonstrating a true AP image. This is visualized by the position of the hemostat clamp which is now shown to be resting underneath the T7 pedicle despite no mechanical manipulation. Thus, the final position of the deployed electrodes is from the inferior aspect of the T8 pedicle to the level of the T10 pedicle. AP, anteroposterior

Once the second electrode is driven into position, we then work to ensure that the end plates of the vertebral bodies are aligned and that the spinous processes are also aligned in a midline orientation. This step can be done initially as well. The electrodes can be tested at this point to assess efficacy/coverage if desired. Afterwards, the Tuohy needles and inner stylets are gently removed without retraction of the electrode wires. Final positioning fluoroscopy is then obtained (Figure 11).

**Figure 12:**
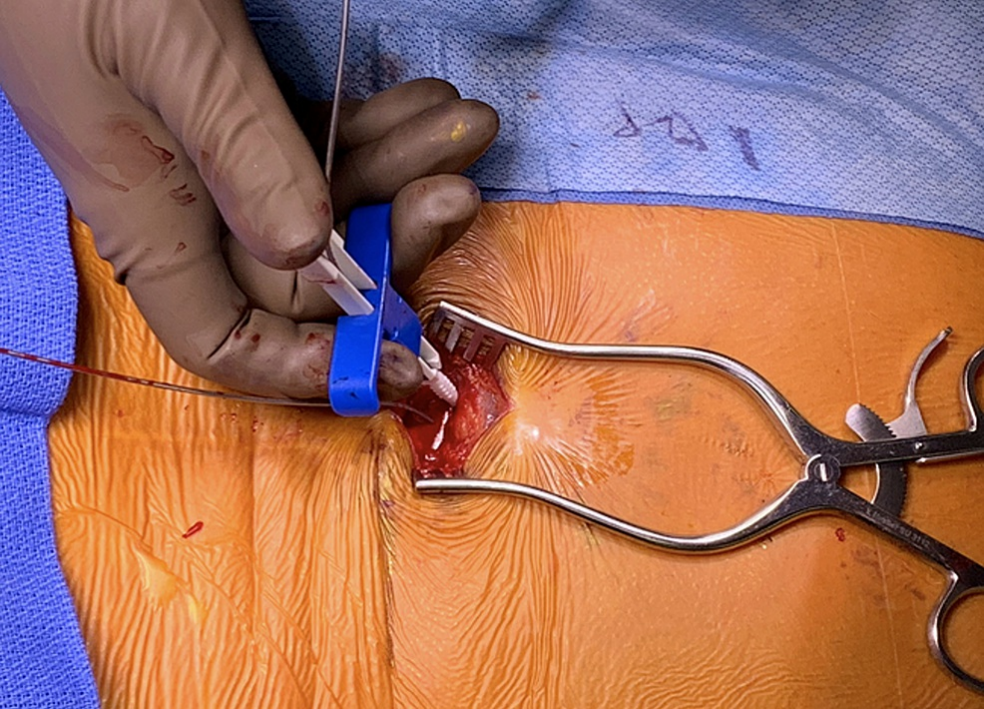
Lead anchoring tool. The provided lead anchoring tool is shown here being deployed at the level of the fascia after the Tuohy needles and inner stylets have been removed. This is done with intermittent fluoroscopy at the final lead location to ensure no movement of the leads during this step.

**Figure 13:**
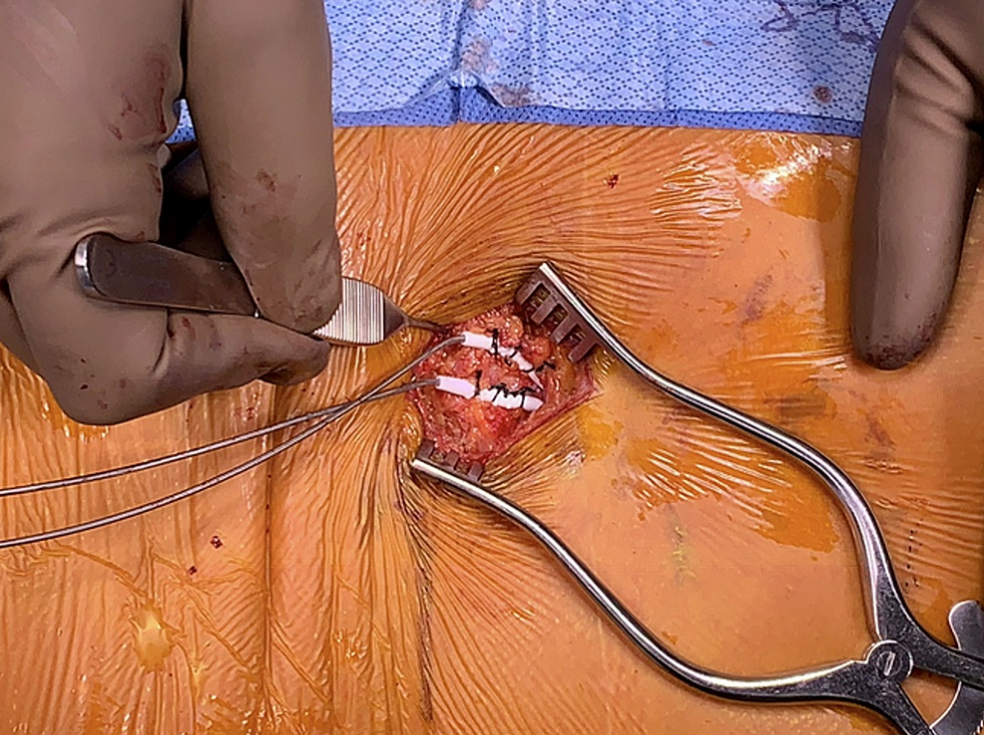
Lead anchoring. Both leads are anchored to the underlying fascia and secured with three non-absorbable sutures each. Intermittent fluoroscopy is also utilized to confirm lead position during the initial anchoring step.

We typically place a self-retaining retractor at this stage to help facilitate anchoring once the leads are visualized at the correct levels. The electrodes are gently withdrawn under fluoroscopy to their target and the anchoring device is then deployed. Intermittent fluoroscopy is utilized to confirm no retraction of the electrodes during this process (Figure 11). Once the anchoring sheath is deployed, multiple non-absorbable sutures are placed directly through the fascia to secure the lead position (Figures 12, 13).

**Figure 14:**
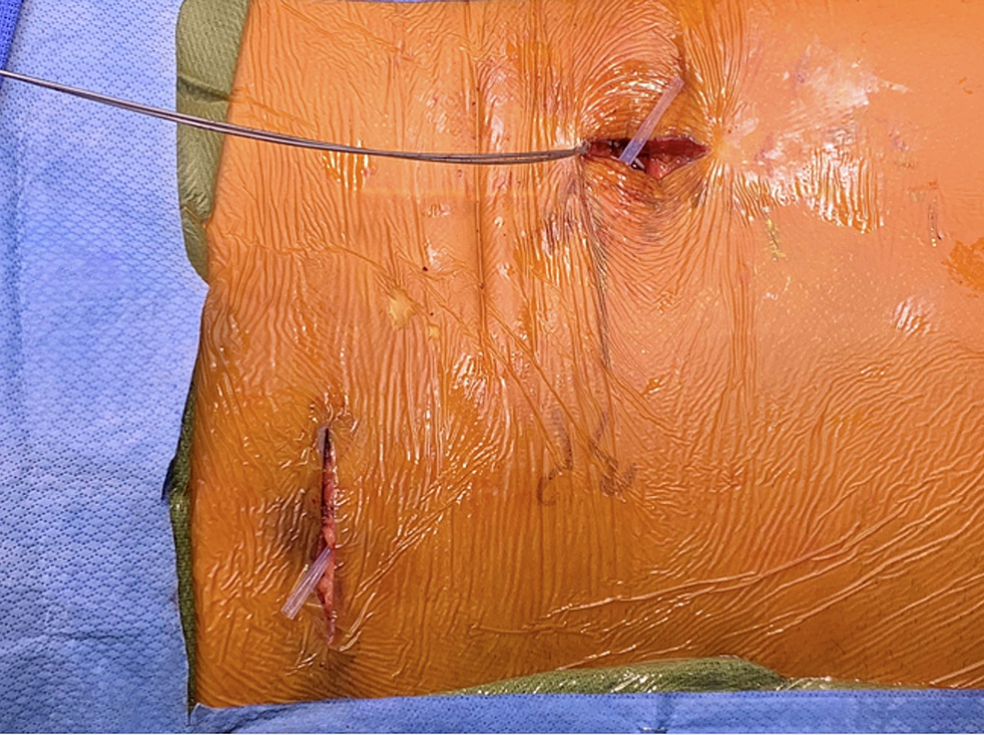
Tunneling. The subcutaneous tunneling tool has been deployed between the spine incision and IPG incision. The plastic sheath is left behind so that the individual percutaneous wires can be passed between both incisions. IPG, implantable pulse generator

The IPG pocket is then created no greater than 2 cm deep from the skin surface. It is advisable to use blunt dissection to create the pocket and ensure that the IPG final resting position is inferior to the incision. Ideally, the incision should not be over the IPG as this can result in post-operative wound dehiscence. The subcutaneous tunneling device is utilized to connect both operative sites (Figure 14).

**Figure 15:**
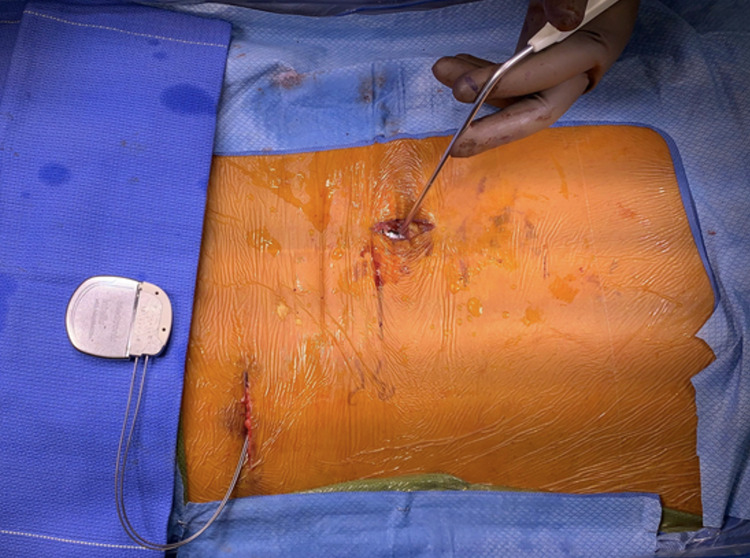
Final placement. The IPG is connected to the electrodes and secured with the locking screws housed in the IPG. The IPG is then placed into the subcutaneous pocket that was created and impedances checked to ensure electrical integrity. IPG, implantable pulse generator

The wires are fed through the plastic sheath and the sheath is then removed. It is good practice to maintain wire laterality when next connecting the leads into the IPG (Figure 15).

The set screws that hold the electrode wires into the IPG are secured with the provided torque screwdriver. The IPG is then placed into the pocket with the extra wire length coiled underneath the IPG. The system is then interrogated for electrical connectivity integrity (impedance check). The IPG is then secured to the deep tissue utilizing non-absorbable sutures passed through built in eyelets on the superior portion of the IPG. The wounds are copiously irrigated with antibiotic irrigation, and a small amount (less than 1 g) of vancomycin powder is instilled in each wound. The deep tissues are closed with absorbable sutures, and the skin is closed with staples, followed by a sterile dressing.

## Discussion

Spinal cord stimulation has become a well-established standard of care for chronic pain syndromes [6]. The percutaneous approach described in this technical report provides a step-wise technique as an alternative to the traditional laminotomy approach. While similar descriptions may be accessed in textbooks, this peer-reviewed manuscript can readily and easily provide novice practitioners with key steps of the procedure. Here, we report the technique of percutaneous thoracic spinal cord stimulator placement that is utilized at our institution.

The patient undergoes standard pre-operative testing, psychological evaluation, and percutaneous lead trial. The trial is often managed by pain management physicians with assistance from the device representative. The most beneficial contact configuration is recorded and the permanent implant is placed to mirror the most effective placement from the trial. Typically, the trial is conducted with superior and inferior broad coverage so that multiple configurations and electrodes can be evaluated. If patients receive greater than 50% improvement in pain, a permanent implant is recommended [5]. A pre-operative thoracic magnetic resonance imaging is obtained prior to permanent lead placement to evaluate for thoracic stenosis, which, if present, could compromise the spinal cord assuming additional mass effect is added to the canal with the procedure. Our practice is to offer percutaneous electrode placement upfront to all patients, with a plan to convert to laminotomy/paddle placement in situations where percutaneous placement is not obtainable intra-operatively.

The percutaneous placement can be performed by interventionalists under local or general anesthesia, whereas the laminotomy approach must be completed by a surgeon [2]. The percutaneous technique has obvious benefits (Table 1): reduced operative times, less exposure to anesthesia, decreased tissue dissection or muscle retraction, improvement in radicular pain, as well as increased durability compared to paddle leads [2,9,10].

**Table 1:** Benefits of the percutaneous technique.

Benefits of the percutaneous technique
Minimally invasive
Cost-effective
Can be placed by interventionalists
Can be performed under local anesthesia
Less operative time and reduced anesthesia
Decreased tissue dissection and muscle retraction
Less mass effect, suitable for some spinal canal abnormalities
Improved durability of leads
Decreased amounts of pain medications post-operatively

Additionally, the overall footprint of the percutaneous electrode is much smaller than the paddle; hence, in cases where patients are noted to have some mild-to-moderate spinal canal stenosis, the percutaneous electrode is much less likely to cause mass effect compared to the paddle [6]. Use of the percutaneous technique allows the implantation of the high-frequency spinal cord stimulator, which was found to be superior for longer periods of coverage and reduced rates of paresthesia compared to the traditional low-frequency spinal cord stimulators [11].

Babu et al. found that patients who underwent the laminotomy (paddle lead) technique had significantly higher complication rates during the initial hospitalization until 90-day follow-up compared to the percutaneous placement alternative. The patients in the paddle group were more likely to develop a post-operative complication (wound infection, pulmonary, neurological sequalae) than patients receiving percutaneous systems. However, they found that the laminotomy (paddle lead) technique required significantly fewer re-operations. Of note, long-term healthcare costs were similar between the two techniques [2].

Limitations of the percutaneous technique must also be considered. The traditional guidewire used for the percutaneous lead placement can increase the risk of dura perforation. Because the steering of the guidewire can be limited due to tracts formed in the fat of the dorsal epidural space, it is recommended that the surgeon ensure under live fluoroscopy the guidewire is correctly inserted into the dorsal epidural space [12]. In addition, percutaneous electrode utilization can present challenges of a higher dislocation rate and higher voltage requirements for the battery [6]. The decision to utilize percutaneous or paddle lead placement can be determined on a case-by-case basis as to which is better suited, thereby highlighting the utility of both approaches.

## Conclusions

Spinal cord stimulation has increased in popularity for the management of chronic pain. A few operative techniques exist in common practice, most commonly the placement of paddle leads placed via laminotomy. While the more invasive laminotomy approach has been well described in the literature, easily accessible detailed techniques of the percutaneous counterpart are lacking. Here, we report the first, readily accessible technical description of the percutaneous placement of thoracic spinal cord stimulator.
